# Cervical ribs—An anatomical obstacle for upper limb regional blocks

**DOI:** 10.1002/ccr3.2467

**Published:** 2019-10-07

**Authors:** Enrique Moreno, Dan Sebastian Dirzu, María Fernanda Bastías Moraga, Mario Fajardo Pérez

**Affiliations:** ^1^ Anesthesia, Regional and Interventional Pain Consultant Dr. Hernan Henríquez Aravena Hospital, Temuco Chile; ^2^ Department of Anesthesia and Intensive Care Emergency County Hospital Cluj Napoca Cluj Napoca România; ^3^ Department of Anesthesia Hospital Universitario de Móstoles Móstoles Spain

**Keywords:** brachial plexus, cervical ribs, supraclavicular block, ultrasound‐guided regional anesthesia

## Abstract

Anatomic variations of the cervical and supraclavicular regions are possible with an impact on regional anesthesia strategy. The presence of cervical ribs may obstruct needle visualization for brachial plexus block in those regions. Preprocedural scan may help in choosing the appropriate technique.

## Graphical abstract

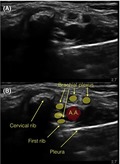

## 1 QUESTION

**Figure 1:**
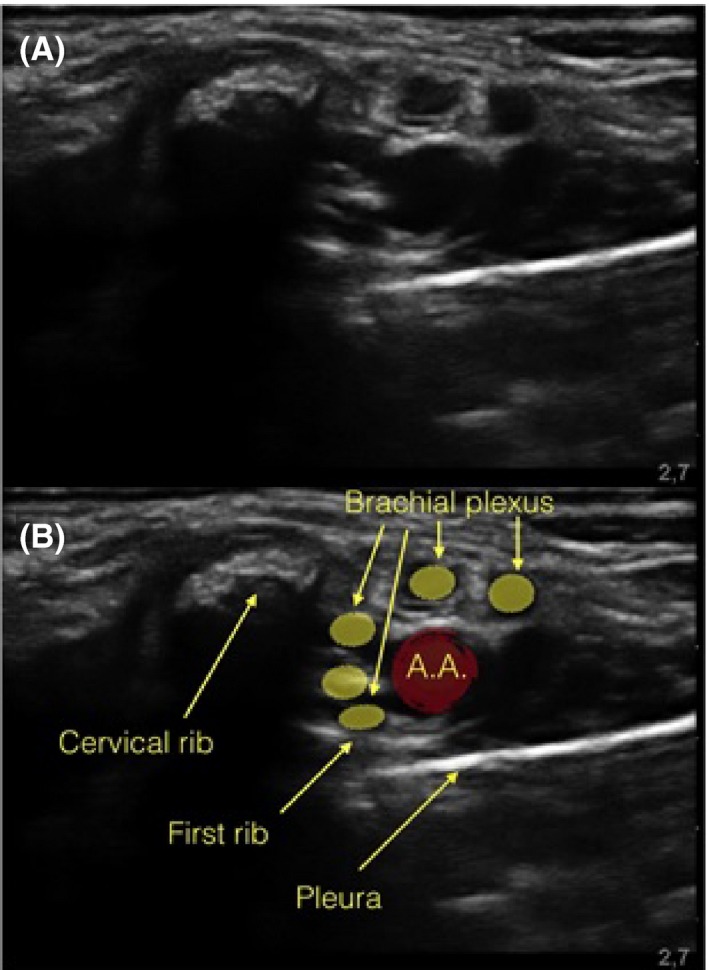
Supraclavicular approach of brachial plexus. A, The ultrasound image of the supraclavicular space. B, The same picture with labels of the anatomical structures AA, subclavian artery

**Figure 2:**
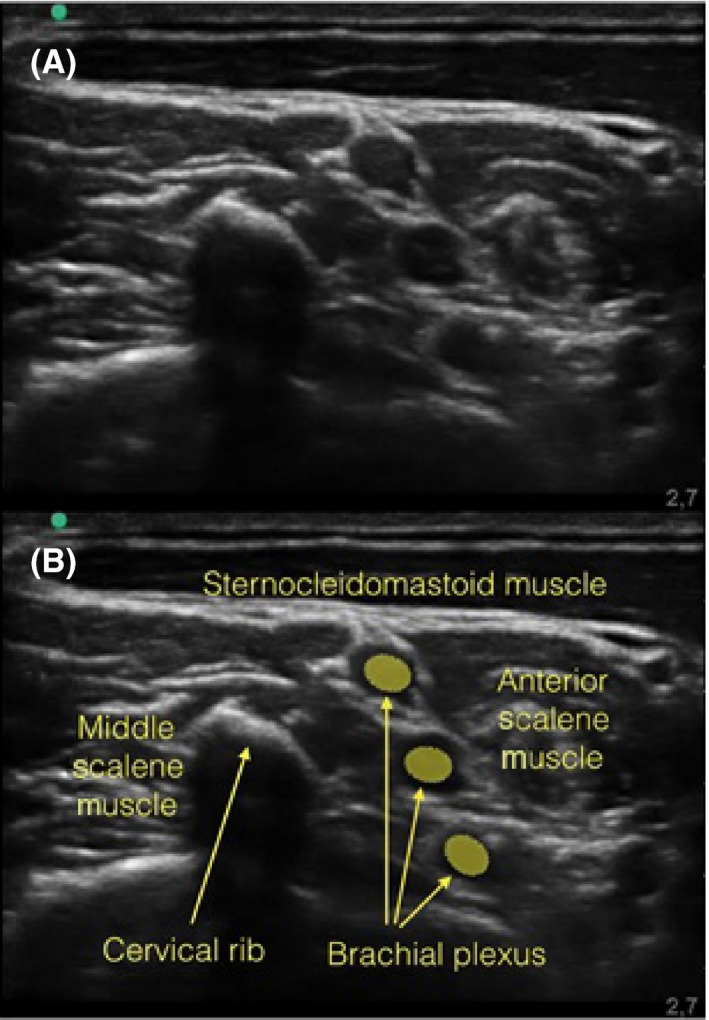
Interscalene approach of brachial plexus. A, The ultrasound of the interscalene space. B, The same picture with labels of the anatomical structures

A 17‐year‐old patient with a left metacarpal fracture was scheduled for an open osteosynthesis. Single shot supraclavicular brachial plexus block was chosen for anesthesia. The skin of the supraclavicular area was prepared and the linear ultrasound probe draped with a sterile sleeve. The scanning in the supraclavicular area was different than expected, with a hyperechoic structure projecting a shadow in the region where the brachial plexus divisions should be (Figure 1). When scanning for interscalene approach, a similar image dividing the middle scalene muscle was noted (Figure 2). Safely targeting the nerves with a needle seemed impossible.

What can it be and what should be done?

## 2 ANSWER

### 2.1 Cervical rib

**Figure 3:**
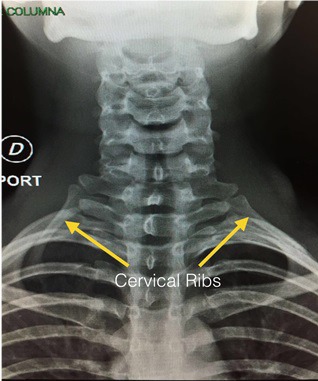
AP X‐ray, yellow arrows show the cervical ribs

Different anatomic variations were described before in relation with performing brachial plexus blocks.^1^, ^2^ Using in‐plane approach and visualizing the entire path of the needle is considered the safest approach considering the presence of sensitive structures in the regions like pleura, blood vessels, and neural foramens. The patient we report presented cervical rib blocking the visualization of the needle path to the brachial plexus in both interscalene and supraclavicular region. An axillary approach was used instead to avoid complications. Anesthesia and surgery were performed uneventful. X‐ray examination performed postoperatively and confirmed the presence of the cervical ribs (Figure 3).

## CONFLICT OF INTEREST

EM, MFB, and MFP have no conflict of interest to disclose related with this paper. DSD is also senior editor of the clinical image section for the Clinical Case Reports journal.

## AUTHOR CONTRIBUTIONS

EM and MFB: involved in patient management, documentation of the case, and writing and reviewing the manuscript. DSD and MFP: involved in writing and reviewing the manuscript.
